# Supplementary material for: Massive Loss of Transcription Factors Promotes the Initial Diversification of Placental Mammals
Source: Int J Mol Sci. 2022 Aug 26;23(17):9720. doi: 10.3390/ijms23179720 (PMC9456351; doi:10.3390/ijms23179720)
Supplement: Supplementary file 1 [file ijms-23-09720-s001.zip › IJMS-1857313-Supplementary Data_final.pdf]

Supplementary Data

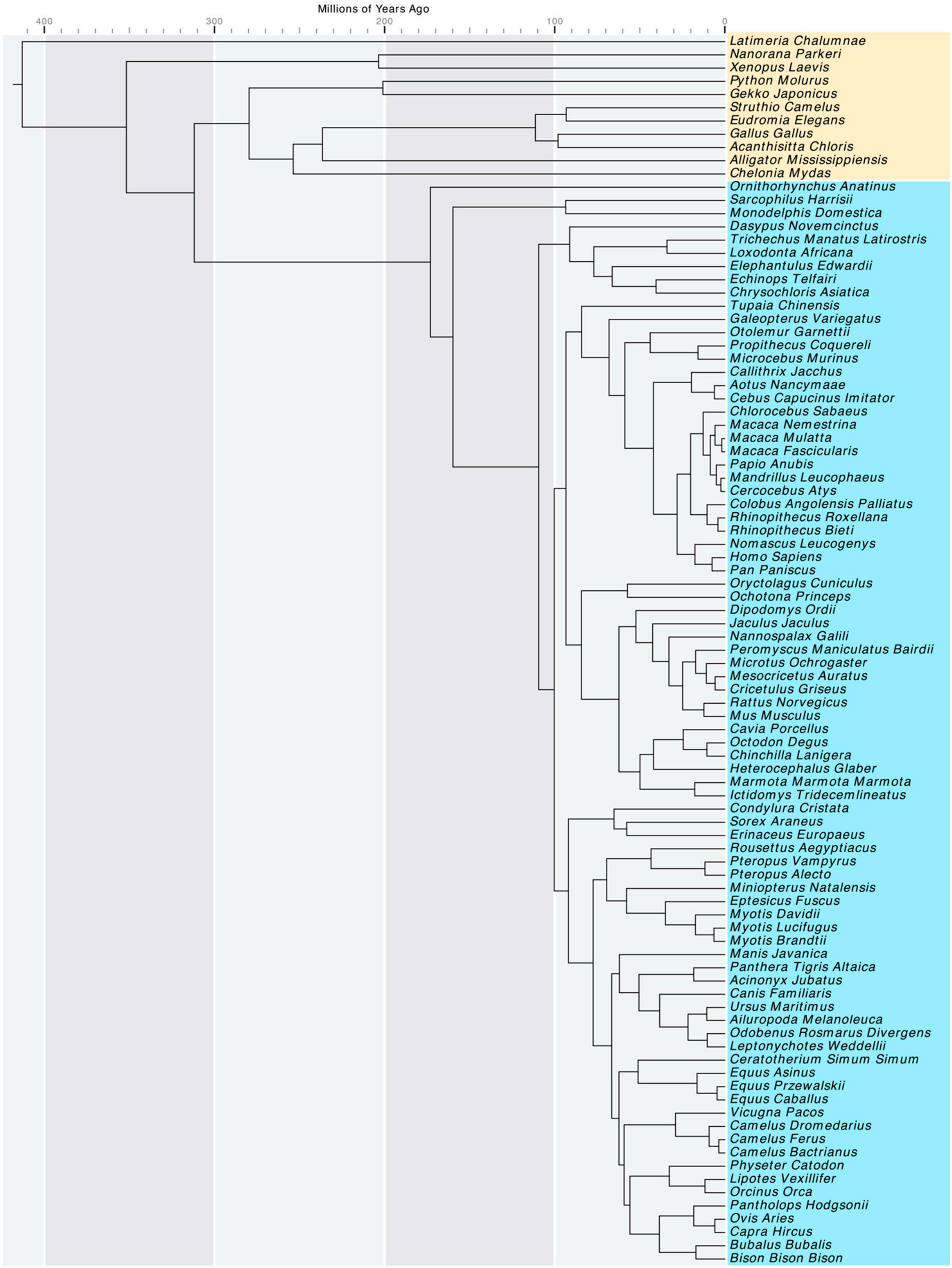

Figure S1. Time tree of 82 mammal species and 11 outgroups. Outgroups with orange background colour. Mammals with blue background colour. Divergence times of outgroups were taken from the Timetree [22] database.

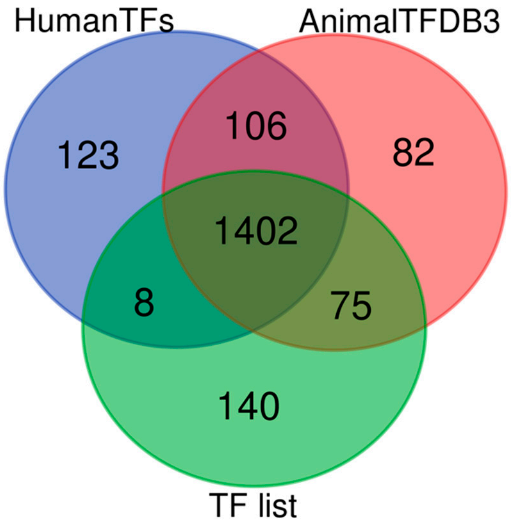

Figure S2. Venn diagram of human transcription factors (TFs) listed in HumanTFs [4], AnimalTFDB3 [45] and our database. Numbers in blue, red and green circles are the number of human TFs in HumanTFs, AnimalTFDB3 and our database, respectively. Numbers in overlapping regions are the number of TFs shared by the different databases.

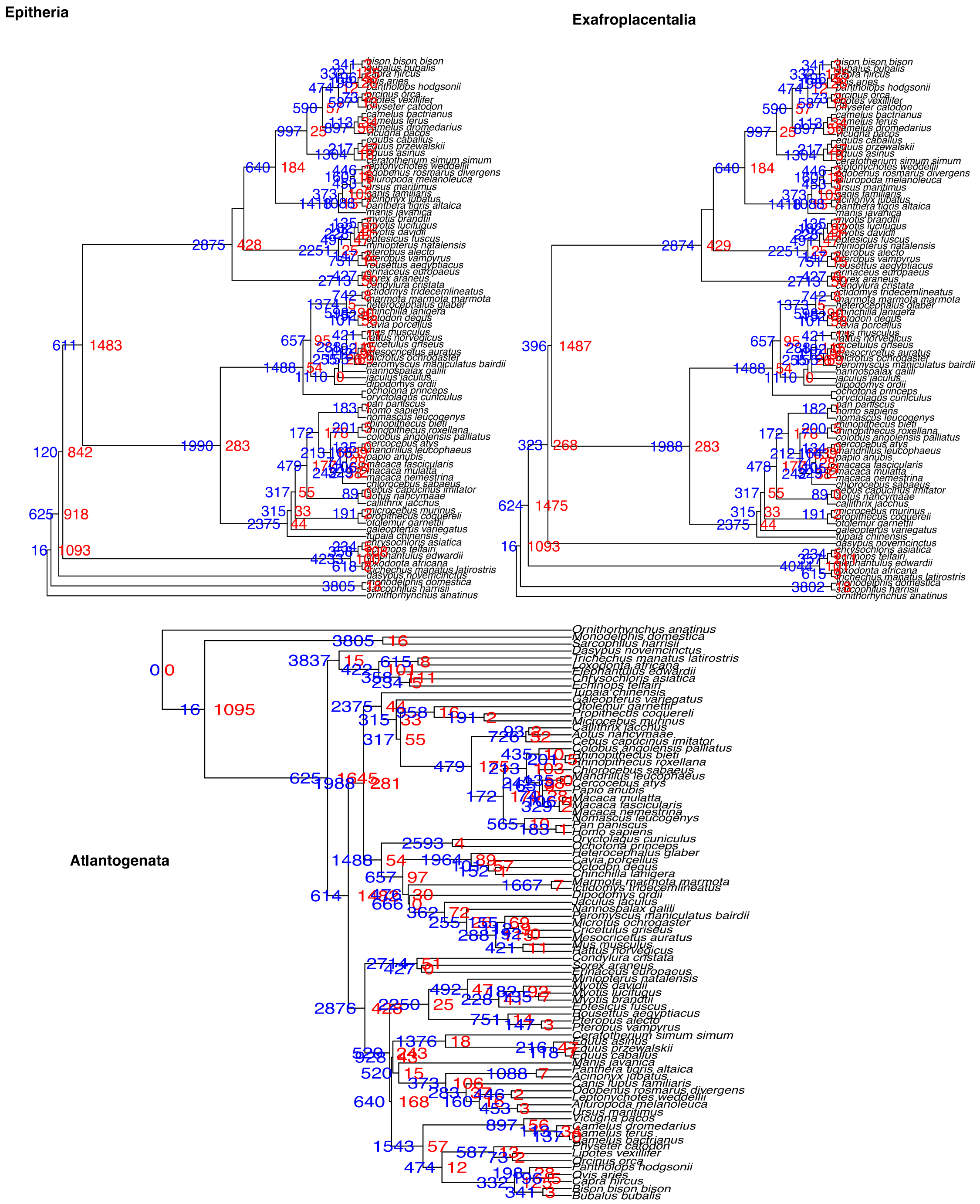

Figure S3. Transcription factor (TF) events in Atlantogenata, Exafroplacentalia and Epitheria inferred using an effective population size of 10^6. Numbers of TF loss and gain events are indicated in blue and red, respectively.

Table S1. Phenotypes of TF genes with significant trait associations.

(a) Diurnality

gene	coefficient	phenotype	PMID
ZNF30	1.176	Chromosome 19q13.11 Deletion Syndrome [57]	25883683
ZNF667 (MIPU1)	0.894	decreased oxLDL-induced cholesterol accumulation [31]	25141035
ZNF648	0.707	high density lipoprotein cholesterol levels [32]	27549350
ZNF836	0.653	Mildly decreased CFP-tsO45G cell surface transport [58]	22660414
ZNF852	0.603	Bartter Syndrome, Type 3 (Ear disease) [59]	27322403
FOXG1	0.598	decreased visual acuity [60]	27001178
ZIM3	0.509	Decreased ionizing radiation sensitivity [61]	20810650
THAP9	0.504	P-element DNA transposase [62]	23349291
HSFX2	0.420	Microsporidiosis [59]	27322403
ZNF311	0.419	Mildly decreased CFP-tsO45G cell surface transport [58]	22660414
ZNF215	0.375	Beckwith-Wiedemann syndrome (overgrowth disorder) [63]	10762538
ZNF534	0.187	Ebstein Anomaly and Patent Foramen Ovale (heart defect) [59]	27322403
FAM186A	0.186	Cerebral Atherosclerosis [64]	29963304
GBX1	0.142	Thermosensory functions [34]	24010020
ZFP92	0.116	Cataract/increased total body fat amount [33]	29092072
ZNF528	0.107	Spiradenoma [65]	28230599
ZNF705G	0.100	Transcriptional regulation [4]	30290144
ZNF558	0.090	decreased circulating glucose level [33]	29092072
ZBTB42	0.062	lethal congenital contracture syndrome [66]	25055871
HES2	0.035	Strongly decreased NFAT1-GFP nuclear translocation [67]	23792561
ZNF33A	0.017	Increased vaccinia virus infection [68]	23401514
ZFP563	-0.010	Anus cancer [69]	30264502
ZFP61 (ZNF135)	-0.022	Neurological deficits [70]	28240725

ZFP868	-0.135	Transcriptional regulation [4]	30290144

(b) Sociality

gene	coefficient	phenotype	PMID
NACC1	4.274	Cerebral atrophy and infantile epilepsy [26]	28132692
SP110	3.928	resistance to tuberculosis [71]	25733846
EPM2A	3.553	Epilepsy [27]	12019206
ZFP112	3.432	Myeloid And Lymphoid Neoplasms Associated With Fgfr1 Abnormalities [72]	22781593
RAB31	3.386	proliferation and apoptosis of cancer cells [73]	22920728
KLF1	3.382	erythropoiesis [74]	21613252
GATAD1	2.706	Gonadotropin secretion [75]	26671628
SRF	2.357	Decreased B or T cell number [76]	17591768
ZFP91	2.060	acute myelogenous leukemia [77]	12738986
ZBED5	1.944	Sindbis virus (SINV) infection [78]	24367265
JUNB	1.936	Chronic myeloid leukemia [79]	11163237
ZNF548	1.643	Increased vaccinia virus infection [68]	23401514
FOXD4	1.208	dilated cardiomyopathy, obsessive-compulsive disorders, and suicidality [80]	17273782
IKZF3	1.093	lenalidomide-induced interleukin-2 production in T cell [81]	24292625
ZNF684	0.677	Increased vaccinia virus infection [68]	23401514
ZNF669	0.634	Increased vaccinia virus infection [68]	23401514
EVX1	0.590	early embryogenesis and neurogenesis [82]	1971786
YBX2	0.496	infertility [83]	18339382
H2BFWT	0.490	Infertility [84]	19583817
ZNF594	0.278	Decreased HIV-1 infection [85]	18854154
ZNF648	0.276	high density lipoprotein cholesterol levels [32]	27549350
ZNF835	0.275	Increased vaccinia virus infection [68]	23401514
SP140	0.210	Innate response to human immunodeficiency virus type 1 [86]	12368356
ZNF780B	0.125	Primary osteoarthritic articular chondrocytes [87]	24976683
LCORL	0.083	body size [88]	23418579
ZNF836	0.063	Mildly decreased CFP-tsO45G cell surface transport [58]	22660414
ZNF84	0.061	cervical cancer [89]	29610946
ZFP563	-0.845	Anus cancer [69]	30264502
ZFP867	-1.421	Transcriptional regulation [4]	30290144
ZNF324	-2.364	uric acid clearance [90]	28095793

(c) Insectivory

gene	coefficient	phenotype	PMID
THAP4	-2.040	Alzheimer's disease [91]	30006735
AFF1	-2.034	Cataract [36]	12629167
ZMIZ2	-0.816	decreased exploration in new environment [33]	29092072
CDX4	-0.811	embryonic hematopoiesis [92]	18511567
HOXA13	-0.494	Hand-foot-genital syndrome [93]	9020844
ZFP57	-0.484	transient neonatal diabetes [94]	18622393
ZNF548	-0.480	Increased vaccinia virus infection [68]	23401514
BHMG1	-0.420	Increased vaccinia virus infection [68]	23401514
ZNF211	-0.411	Decreased circadian period length [95]	19765810
ZSCAN30 (ZNF397OS)	-0.390	obese [96]	29717273
SEBOX	-0.375	increased circulating total protein level/glucose tolerance [33]	29092072
NSL1	-0.373	increased circulating HDL cholesterol level/corneal opacity [33]	29092072
TULP1	-0.340	autosomal recessive retinitis pigmentosa [97]	9462751
ZNF648	-0.288	high density lipoprotein cholesterol levels [32]	27549350
ZNF419	-0.263	Novel Renal Cell [99]	21738768
PASD1	-0.189	circadian clock [100]	25936801
ISL2	-0.185	pre-laminated chick retina [101]	16864127
PHOX2A	-0.141	improved glucose tolerance [35]	11343120
BARX1	-0.081	abnormal tooth development [102]	22084104
ZNF860	-0.049	Increased vaccinia virus infection [68]	23401514
KLF8	-0.024	increased circulating total protein level [33]	29092072

(d) Reproductive seasonality

gene	coefficient	phenotype	PMID
DLX4	-1.797	fetal growth restriction [103]	17062780
ZFP184	-1.775	abnormal behavior [33]	29092072
TMEM185A	-1.657	Fraxf Syndrome [59]	27322403
ZNF727	-1.515	Transcriptional regulation [4]	30290144
BARHL1	-1.487	Hearing loss [104]	15044550
ZFP94	-1.435	Transcriptional regulation [4]	30290144
ZFP407	-1.252	failure of blastocyst formation [33]	29092072
ZFP560	-1.049	Decreased viability [105]	29141986
ZNF749	-0.861	Increased viability [106]	25170077
ZNF180	-0.801	Decreased viability [105]	29141986
TPRX1	-0.737	early embryonic development [59]	27322403
RHOX4G	-0.626	embryonic stem cell [107]	16916441

ZFP59	-0.592	accumulates in spermatozoa nuclei [108]	8547218
HSFX2	-0.338	Microsporidiosis [59]	27322403
MESP1	-0.219	embryonic growth retardation [109]	9739106
ESX1	-0.210	fetal growth retardation [110]	9806555
ZSCAN12	-0.169	abnormal bone structure [33]	29092072
ZNF232	-0.156	Decreased viability [111]	24442637
HOXA13	-0.017	Hand-foot-genital syndrome [93]	9020844
ZFP654	-0.005	Decreased viability [105]	29141986
ZNF518A	1.875	Increased vaccinia virus infection [68]	23401514
ZNF524	1.944	Increased viability [106]	25170077
ZNF414	2.909	Decreased viability [105]	29141986

References

32. Beaney, K.E.; Cooper, J.A.; McLachlan, S.; Wannamethee, S.G.; Jefferis, B.J.; Whincup, P.; Ben-Shlomo, Y.; Price, J.F.; Kumari, M.; Wong, A.; et al. Variant rs10911021 that associates with coronary heart disease in type 2 diabetes, is associated with lower concentrations of circulating HDL cholesterol and large HDL particles but not with amino acids. *Cardiovasc Diabetol* **2016**, *15*, 115, doi:10.1186/s12933-016-0435-0.
57. Melo, J.B.; Estevinho, A.; Saraiva, J.; Ramos, L.; Carreira, I.M. Cutis Aplasia as a clinical hallmark for the syndrome associated with 19q13.11 deletion: the possible role for UBA2 gene. *Mol Cytogenet* **2015**, *8*, 21, doi:10.1186/s13039-015-0123-x.
58. Simpson, J.C.; Joggerst, B.; Laketa, V.; Verissimo, F.; Cetin, C.; Erfle, H.; Bexiga, M.G.; Singan, V.R.; Heriche, J.K.; Neumann, B.; et al. Genome-wide RNAi screening identifies human proteins with a regulatory function in the early secretory pathway. *Nat Cell Biol* **2012**, *14*, 764-774, doi:10.1038/ncb2510.
59. Stelzer, G.; Rosen, N.; Plaschkes, I.; Zimmerman, S.; Twik, M.; Fishilevich, S.; Stein, T.I.; Nudel, R.; Lieder, I.; Mazor, Y.; et al. The GeneCards Suite: From Gene Data Mining to Disease Genome Sequence Analyses. *Curr Protoc Bioinformatics* **2016**, *54*, 1 30 31-31 30 33, doi:10.1002/cpbi.5.
60. Boggio, E.M.; Pancrazi, L.; Gennaro, M.; Lo Rizzo, C.; Mari, F.; Meloni, I.; Ariani, F.; Panighini, A.; Novelli, E.; Biagioni, M.; et al. Visual impairment in FOXG1-mutated individuals and mice. *Neuroscience* **2016**, *324*, 496-508, doi:10.1016/j.neuroscience.2016.03.027.
61. Hurov, K.E.; Cotta-Ramusino, C.; Elledge, S.J. A genetic screen identifies the Triple T complex required for DNA damage signaling and ATM and ATR stability. *Genes Dev* **2010**, *24*, 1939-1950, doi:10.1101/gad.1934210.
62. Majumdar, S.; Singh, A.; Rio, D.C. The human THAP9 gene encodes an active P-element DNA transposase. *Science* **2013**, *339*, 446-448, doi:10.1126/science.1231789.
63. Alders, M.; Ryan, A.; Hodges, M.; Bliek, J.; Feinberg, A.P.; Privitera, O.; Westerveld, A.; Little, P.F.; Mannens, M. Disruption of a novel imprinted zinc-finger gene, ZNF215, in Beckwith-Wiedemann syndrome. *Am J Hum Genet* **2000**, *66*, 1473-1484, doi:10.1086/302892.
64. Yasukochi, Y.; Sakuma, J.; Takeuchi, I.; Kato, K.; Oguri, M.; Fujimaki, T.; Horibe, H.; Yamada, Y. Six novel susceptibility loci for coronary artery disease and cerebral infarction identified by longitudinal exome-wide association studies in a Japanese population. *Biomed Rep* **2018**, *9*, 123-134, doi:10.3892/br.2018.1109.
65. Liu, L.; Bu, H.; Yang, Y.; Tan, Z.; Zhang, F.; Hu, S.; Zhao, T. A Targeted, Next-Generation Genetic Sequencing Study on Tetralogy of Fallot, Combined With Cleft Lip and Palate. *J Craniofac Surg* **2017**, *28*, e351-e355, doi:10.1097/SCS.0000000000003598.
66. Patel, N.; Smith, L.L.; Faqeih, E.; Mohamed, J.; Gupta, V.A.; Alkuraya, F.S. ZBTB42 mutation defines a novel lethal congenital contracture syndrome (LCCS6). *Hum Mol Genet* **2014**, *23*, 6584-6593, doi:10.1093/hmg/ddu384.
67. Sharma, S.; Quintana, A.; Findlay, G.M.; Mettlen, M.; Baust, B.; Jain, M.; Nilsson, R.; Rao, A.; Hogan, P.G. An siRNA screen for NFAT activation identifies septins as coordinators of store-operated Ca2+ entry. *Nature* **2013**, *499*, 238-242, doi:10.1038/nature12229.

68. Sivan, G.; Martin, S.E.; Myers, T.G.; Buehler, E.; Szymczyk, K.H.; Ormanoglu, P.; Moss, B. Human genome-wide RNAi screen reveals a role for nuclear pore proteins in poxvirus morphogenesis. *Proc Natl Acad Sci U S A* **2013**, *110*, 3519-3524, doi:10.1073/pnas.1300708110.
69. Jeannot, E.; Harle, A.; Holmes, A.; Sastre-Garau, X. Nuclear factor I X is a recurrent target for HPV16 insertions in anal carcinomas. *Genes Chromosomes Cancer* **2018**, *57*, 638-644, doi:10.1002/gcc.22675.
70. Raghuram, V.; Weber, S.; Raber, J.; Chen, D.H.; Bird, T.D.; Maylie, J.; Adelman, J.P. Assessment of mutations in KCNN2 and ZNF135 to patient neurological symptoms. *Neuroreport* **2017**, *28*, 375-379, doi:10.1097/WNR.0000000000000754.
71. Wu, H.; Wang, Y.; Zhang, Y.; Yang, M.; Lv, J.; Liu, J.; Zhang, Y. TALE nickase-mediated SP110 knockin endows cattle with increased resistance to tuberculosis. *Proc Natl Acad Sci U S A* **2015**, *112*, E1530-1539, doi:10.1073/pnas.1421587112.
72. Ren, M.; Qin, H.; Ren, R.; Cowell, J.K. Ponatinib suppresses the development of myeloid and lymphoid malignancies associated with FGFR1 abnormalities. *Leukemia* **2013**, *27*, 32-40, doi:10.1038/leu.2012.188.
73. Grismayer, B.; Solch, S.; Seubert, B.; Kirchner, T.; Schafer, S.; Baretton, G.; Schmitt, M.; Luther, T.; Kruger, A.; Kotzsch, M.; et al. Rab31 expression levels modulate tumor-relevant characteristics of breast cancer cells. *Mol Cancer* **2012**, *11*, 62, doi:10.1186/1476-4598-11-62.
74. Siatecka, M.; Bieker, J.J. The multifunctional role of EKLF/KLF1 during erythropoiesis. *Blood* **2011**, *118*, 2044-2054, doi:10.1182/blood-2011-03-331371.
75. Lomniczi, A.; Wright, H.; Castellano, J.M.; Matagne, V.; Toro, C.A.; Ramaswamy, S.; Plant, T.M.; Ojeda, S.R. Epigenetic regulation of puberty via Zinc finger protein-mediated transcriptional repression. *Nat Commun* **2015**, *6*, 10195, doi:10.1038/ncomms10195.
76. Fleige, A.; Alberti, S.; Grobe, L.; Frischmann, U.; Geffers, R.; Muller, W.; Nordheim, A.; Schippers, A. Serum response factor contributes selectively to lymphocyte development. *J Biol Chem* **2007**, *282*, 24320-24328, doi:10.1074/jbc.M703119200.
77. Unoki, M.; Okutsu, J.; Nakamura, Y. Identification of a novel human gene, ZFP91, involved in acute myelogenous leukemia. *Int J Oncol* **2003**, *22*, 1217-1223.
78. Ooi, Y.S.; Stiles, K.M.; Liu, C.Y.; Taylor, G.M.; Kielian, M. Genome-wide RNAi screen identifies novel host proteins required for alphavirus entry. *PLoS Pathog* **2013**, *9*, e1003835, doi:10.1371/journal.ppat.1003835.
79. Passegue, E.; Jochum, W.; Schorpp-Kistner, M.; Mohle-Steinlein, U.; Wagner, E.F. Chronic myeloid leukemia with increased granulocyte progenitors in mice lacking junB expression in the myeloid lineage. *Cell* **2001**, *104*, 21-32, doi:10.1016/s0092-8674(01)00188-x.
80. Minoretti, P.; Arra, M.; Emanuele, E.; Olivieri, V.; Aldeghi, A.; Politi, P.; Martinelli, V.; Pesenti, S.; Falcone, C. A W148R mutation in the human FOXD4 gene segregating with dilated cardiomyopathy, obsessive-compulsive disorder, and suicidality. *Int J Mol Med* **2007**, *19*, 369-372.
81. Kronke, J.; Udeshi, N.D.; Narla, A.; Grauman, P.; Hurst, S.N.; McConkey, M.; Svinkina, T.; Heckl, D.; Comer, E.; Li, X.; et al. Lenalidomide causes selective degradation of IKZF1 and IKZF3 in multiple myeloma cells. *Science* **2014**, *343*, 301-305, doi:10.1126/science.1244851.
82. Bastian, H.; Gruss, P. A murine even-skipped homologue, Evx 1, is expressed during early embryogenesis and neurogenesis in a biphasic manner. *EMBO J* **1990**, *9*, 1839-1852, doi:10.1002/j.1460-2075.1990.tb08309.x.
83. Hammoud, S.; Emery, B.R.; Dunn, D.; Weiss, R.B.; Carrell, D.T. Sequence alterations in the YBX2 gene are associated with male factor infertility. *Fertil Steril* **2009**, *91*, 1090-1095, doi:10.1016/j.fertnstert.2008.01.009.
84. Lee, J.; Park, H.S.; Kim, H.H.; Yun, Y.J.; Lee, D.R.; Lee, S. Functional polymorphism in H2BFWT-5'UTR is associated with susceptibility to male infertility. *J Cell Mol Med* **2009**, *13*, 1942-1951, doi:10.1111/j.1582-4934.2009.00830.x.
85. Konig, R.; Zhou, Y.; Elleder, D.; Diamond, T.L.; Bonamy, G.M.; Irelan, J.T.; Chiang, C.Y.; Tu, B.P.; De Jesus, P.D.; Lilley, C.E.; et al. Global analysis of host-pathogen interactions that regulate early-stage HIV-1 replication. *Cell* **2008**, *135*, 49-60, doi:10.1016/j.cell.2008.07.032.
86. Madani, N.; Millette, R.; Platt, E.J.; Marin, M.; Kozak, S.L.; Bloch, D.B.; Kabat, D. Implication of the lymphocyte-specific nuclear body protein Sp140 in an innate response to human

immunodeficiency virus type 1. *J Virol* **2002**, *76*, 11133-11138, doi:10.1128/jvi.76.21.11133-11138.2002.
87. Mesuraca, M.; Galasso, O.; Guido, L.; Chiarella, E.; Scicchitano, S.; Vatrinet, R.; Morrone, G.; Bond, H.M.; Gasparini, G. Expression profiling and functional implications of a set of zinc finger proteins, ZNF423, ZNF470, ZNF521, and ZNF780B, in primary osteoarthritic articular chondrocytes. *Mediators Inflamm* **2014**, *2014*, 318793, doi:10.1155/2014/318793.
88. Metzger, J.; Schrimpf, R.; Philipp, U.; Distl, O. Expression levels of LCORL are associated with body size in horses. *PLoS One* **2013**, *8*, e56497, doi:10.1371/journal.pone.0056497.
89. Li, P.; Guo, H.; Zhou, G.; Shi, H.; Li, Z.; Guan, X.; Deng, Z.; Li, S.; Zhou, S.; Wang, Y.; et al. Increased ZNF84 expression in cervical cancer. *Arch Gynecol Obstet* **2018**, *297*, 1525-1532, doi:10.1007/s00404-018-4770-0.
90. Chittoor, G.; Haack, K.; Mehta, N.R.; Laston, S.; Cole, S.A.; Comuzzie, A.G.; Butte, N.F.; Voruganti, V.S. Genetic variation underlying renal uric acid excretion in Hispanic children: the Viva La Familia Study. *BMC Med Genet* **2017**, *18*, 6, doi:10.1186/s12881-016-0366-3.
91. Yamaguchi-Kabata, Y.; Morihara, T.; Ohara, T.; Ninomiya, T.; Takahashi, A.; Akatsu, H.; Hashizume, Y.; Hayashi, N.; Shigemizu, D.; Boroevich, K.A.; et al. Integrated analysis of human genetic association study and mouse transcriptome suggests LBH and SHF genes as novel susceptible genes for amyloid-beta accumulation in Alzheimer's disease. *Hum Genet* **2018**, *137*, 521-533, doi:10.1007/s00439-018-1906-z.
92. Wang, Y.; Yabuuchi, A.; McKinney-Freeman, S.; Ducharme, D.M.; Ray, M.K.; Chawengsaksophak, K.; Archer, T.K.; Daley, G.Q. Cdx gene deficiency compromises embryonic hematopoiesis in the mouse. *Proc Natl Acad Sci U S A* **2008**, *105*, 7756-7761, doi:10.1073/pnas.0708951105.
93. Mortlock, D.P.; Innis, J.W. Mutation of HOXA13 in hand-foot-genital syndrome. *Nat Genet* **1997**, *15*, 179-180, doi:10.1038/ng0297-179.
94. Mackay, D.J.; Callaway, J.L.; Marks, S.M.; White, H.E.; Acerini, C.L.; Boonen, S.E.; Dayanikli, P.; Firth, H.V.; Goodship, J.A.; Haemers, A.P.; et al. Hypomethylation of multiple imprinted loci in individuals with transient neonatal diabetes is associated with mutations in ZFP57. *Nat Genet* **2008**, *40*, 949-951, doi:10.1038/ng.187.
95. Zhang, E.E.; Liu, A.C.; Hirota, T.; Miraglia, L.J.; Welch, G.; Pongsawakul, P.Y.; Liu, X.; Atwood, A.; Huss, J.W., 3rd; Janes, J.; et al. A genome-wide RNAi screen for modifiers of the circadian clock in human cells. *Cell* **2009**, *139*, 199-210, doi:10.1016/j.cell.2009.08.031.
96. Crujeiras, A.B.; Morcillo, S.; Diaz-Lagares, A.; Sandoval, J.; Castellano-Castillo, D.; Torres, E.; Hervas, D.; Moran, S.; Esteller, M.; Macias-Gonzalez, M.; et al. Identification of an episignature of human colorectal cancer associated with obesity by genome-wide DNA methylation analysis. *Int J Obes (Lond)* **2019**, *43*, 176-188, doi:10.1038/s41366-018-0065-6.
97. Banerjee, P.; Kleyn, P.W.; Knowles, J.A.; Lewis, C.A.; Ross, B.M.; Parano, E.; Kovats, S.G.; Lee, J.J.; Penchaszadeh, G.K.; Ott, J.; et al. TULP1 mutation in two extended Dominican kindreds with autosomal recessive retinitis pigmentosa. *Nat Genet* **1998**, *18*, 177-179, doi:10.1038/ng0298-177.
98. Broen, K.; Levenga, H.; Vos, J.; van Bergen, K.; Fredrix, H.; Greupink-Draaisma, A.; Kester, M.; Falkenburg, J.H.; de Mulder, P.; de Witte, T.; et al. A polymorphism in the splice donor site of ZNF419 results in the novel renal cell carcinoma-associated minor histocompatibility antigen ZAPHIR. *PLoS One* **2011**, *6*, e21699, doi:10.1371/journal.pone.0021699.
99. Michael, A.K.; Harvey, S.L.; Sammons, P.J.; Anderson, A.P.; Kopalle, H.M.; Banham, A.H.; Partch, C.L. Cancer/Testis Antigen PASD1 Silences the Circadian Clock. *Mol Cell* **2015**, *58*, 743-754, doi:10.1016/j.molcel.2015.03.031.
100. Edqvist, P.H.; Myers, S.M.; Hallbook, F. Early identification of retinal subtypes in the developing, pre-laminated chick retina using the transcription factors Prox1, Lim1, Ap2alpha, Pax6, Isl1, Isl2, Lim3 and Chx10. Eur J Histochem 2006, 50, 147-154.
101. Miletich, I.; Yu, W.Y.; Zhang, R.; Yang, K.; Caixeta de Andrade, S.; Pereira, S.F.; Ohazama, A.; Mock, O.B.; Buchner, G.; Sealby, J.; et al. Developmental stalling and organ-autonomous regulation of morphogenesis. Proc Natl Acad Sci U S A 2011, 108, 19270-19275, doi:10.1073/pnas.1112801108.

102. Murthi, P.; Said, J.M.; Doherty, V.L.; Donath, S.; Nowell, C.J.; Brennecke, S.P.; Kalionis, B. Homeobox gene DLX4 expression is increased in idiopathic human fetal growth restriction. Mol Hum Reprod 2006, 12, 763-769, doi:10.1093/molehr/gal087.
103. Li, S.; Qiu, F.; Xu, A.; Price, S.M.; Xiang, M. Barhl1 regulates migration and survival of cerebellar granule cells by controlling expression of the neurotrophin-3 gene. J Neurosci 2004, 24, 3104-3114, doi:10.1523/JNEUROSCI.4444-03.2004.
104. Song, O.R.; Queval, C.J.; Iantomasi, R.; Delorme, V.; Marion, S.; Veyron-Churlet, R.; Werkmeister, E.; Popoff, M.; Ricard, I.; Jouny, S.; et al. ArfGAP1 restricts Mycobacterium tuberculosis entry by controlling the actin cytoskeleton. EMBO Rep 2018, 19, 29-42, doi:10.15252/embr.201744371.
105. Warner, N.; Burberry, A.; Pliakas, M.; McDonald, C.; Nunez, G. A genome-wide small interfering RNA (siRNA) screen reveals nuclear factor-kappaB (NF-kappaB)-independent regulators of NOD2-induced interleukin-8 (IL-8) secretion. J Biol Chem 2014, 289, 28213-28224, doi:10.1074/jbc.M114.574756.
106. Jackson, M.; Watt, A.J.; Gautier, P.; Gilchrist, D.; Driehaus, J.; Graham, G.J.; Keebler, J.; Prugnolle, F.; Awadalla, P.; Forrester, L.M. A murine specific expansion of the Rhox cluster involved in embryonic stem cell biology is under natural selection. BMC Genomics 2006, 7, 212, doi:10.1186/1471-2164-7-212.
107. Passananti, C.; Corbi, N.; Paggi, M.G.; Russo, M.A.; Perez, M.; Cotelli, F.; Stefanini, M.; Amati, P. The product of Zfp59 (Mfg2), a mouse gene expressed at the spermatid stage of spermatogenesis, accumulates in spermatozoa nuclei. Cell Growth Differ 1995, 6, 1037-1044.
108. Saga, Y. Genetic rescue of segmentation defect in MesP2-deficient mice by MesP1 gene replacement. Mech Dev 1998, 75, 53-66, doi:10.1016/s0925-4773(98)00077-x.
109. Li, Y.; Behringer, R.R. Esx1 is an X-chromosome-imprinted regulator of placental development and fetal growth. Nat Genet 1998, 20, 309-311, doi:10.1038/3129.
110. Kranz, D.; Boutros, M. A synthetic lethal screen identifies FAT1 as an antagonist of caspase-8 in extrinsic apoptosis. EMBO J 2014, 33, 181-197, doi:10.1002/embj.201385686.
